# Gestational diabetes mellitus is associated with the neonatal gut microbiota and metabolome

**DOI:** 10.1186/s12916-021-01991-w

**Published:** 2021-05-27

**Authors:** Ting Chen, Yufeng Qin, Minjian Chen, Yuqing Zhang, Xu Wang, Tianyu Dong, Guanglin Chen, Xian Sun, Ting Lu, Richard Allen White, Peng Ye, Hein M. Tun, Yankai Xia

**Affiliations:** 1 Women’s Hospital of Nanjing Medical University, Nanjing Maternity and Child Health Care Hospital, Nanjing, 210004 China; 2 Department of Microbes and Infection, School of Public Health, Nanjing Medical University, Nanjing, 211166 China; 3 State Key Laboratory of Reproductive Medicine, Institute of Toxicology, School of Public Health, Nanjing Medical University, Nanjing, 211166 China; 4 Key Laboratory of Modern Toxicology of Ministry of Education, School of Public Health, Nanjing Medical University, Nanjing, 211166 China; 5 Department of Endocrinology, Children’s Hospital of Nanjing Medical University, Nanjing, 210000 China; 6 Department of Pediatric Surgery, Children’s Hospital of Nanjing Medical University, Nanjing, 210000 China; 7 Department of Bioinformatics and Genomics at University of North Carolina, Charlotte, USA; 8 RAW Molecular Systems LLC, Concord, USA; 9 HKU-Pasteur Research Pole, School of Public Health, Li Ka Shing Faculty of Medicine, The University of Hong Kong, Hong Kong, SAR China

**Keywords:** Gestational diabetes mellitus, Microbiota, Metabolome

## Abstract

**Background:**

Gestational diabetes mellitus (GDM) is a metabolic disease that occurs in pregnant women and increases the risk for the development of diabetes. The relationship between GDM and meconium microbiota and metabolome remains incompletely understood.

**Methods:**

Four hundred eighteen mothers (147 women with GDM and 271 normal pregnant women) and their neonates from the GDM Mother and Child Study were included in this study. Meconium microbiota were profiled by 16S rRNA gene sequencing. Meconium and maternal serum metabolome were examined by UPLC-QE.

**Results:**

Microbial communities in meconium were significantly altered in neonates from the GDM mothers. A reduction in alpha diversity was observed in neonates of GDM mothers. At the phylum level, the abundance of *Firmicutes* and *Proteobacteria* changed significantly in neonates of GDM mothers. Metabolomic analysis of meconium showed that metabolic pathways including taurine and hypotaurine metabolism, pyrimidine metabolism, beta-alanine metabolism, and bile acid biosynthesis were altered in GDM subjects. Several changed metabolites varying by the similar trend across the maternal serum and neonatal meconium were observed.

**Conclusion:**

Altogether, these findings suggest that GDM could alter the serum metabolome and is associated with the neonatal meconium microbiota and metabolome, highlighting the importance of maternal factors on early-life metabolism.

**Supplementary Information:**

The online version contains supplementary material available at 10.1186/s12916-021-01991-w.

## Background

Gestational diabetes mellitus (GDM) is defined as any degree of glucose intolerance with an onset or first recognition during pregnancy, which is one of the most common types of pregnancy complications [1]. Women with GDM are more susceptible to other pregnancy complications including pre-eclampsia, preterm delivery, and metabolic syndrome. Besides that, GDM increases not only the risk of fetal macrosomia, neonatal hypoglycemia, jaundice, polycythemia, and hypocalcemia during the perinatal period, but also the risk of developing childhood obesity and metabolic syndrome later in life [2–4]. Although numerous studies have been done to explore the potential connections between GDM mothers and long-term consequences on their children, it still has not been fully understood.

The human body harbors trillions of microbial cells and they are indispensable for human health. The gut microbiota resides on the intestinal mucosal surfaces and participates in epithelial homeostasis, energy harvest, and immune development [5, 6]. Colonization of the infant’s gut has drawn great interest, because it links to individual’s health and late-onset diseases [7–9]. Lots of efforts have been made to understand the gut microbiota and its function in early infancy. However, factors that affect neonatal gut microbiota and metabolome are remained incompletely understood [10].

Microorganisms in meconium were the first colonizers of the newborn, which come from the mother’s skin, vagina, and gut [7]. A wide variety of reports had demonstrated that microbiotain meconium could be affected by the delivery mode, perinatal antibiotics, and breastfeeding [11, 12]. Wang et al. showed that GDM altered the microbial community in meconium from neonates delivered by C-section, with a similar trend in maternal gut microbiota changes [13]. Besides microbes or their structural components, microbial metabolites were also important to host physiology [14, 15], but it has been barely studied in meconium [16]. Therefore, an integrated analysis of the maternal metabolome and the neonatal meconium microbiota and metabolome may provide a comprehensive understanding of the impact of GDM on microbial colonization in early life.

## Methods

### Study design and participants

Mothers and their neonates in the GDM Mother and Child Study (GMCS) were recruited at Women’s Hospital of Nanjing Medical University (Nanjing, Jiangsu Province, China). The study was approved by the Medicine Ethics Committee at Women’s Hospital of Nanjing Medical University (IRB Number: [2016]009). All participants provided written informed consent for themselves and the neonates. All participants were offered a standardized 75-g oral glucose tolerance test (OGTT) between 25 and 26 weeks during pregnancy. Women with GDM were diagnosed by qualified doctors if one or more of the following glucose criteria were met: fasting ≥ 5.1 mmol/L, 1 h ≥ 10.0 mmol/L, or 2 h ≥ 8.5 mmol/L [17]. A structured questionnaire was used to collect demographic information, as well as information of potential risk factors including age, pre-pregnancy body mass index (BMI), abnormal pregnancy history, and family history of diabetes. Additionally, the gestational age, delivery mode, gender, and birth weight were extracted from a hospital computer-based patient record (CPR) information system. In this study, subjects with pre-existing diabetes, pre-existing metabolic diseases, antibiotics usage within 3 months, alcohol or substance abuse, and chronic diseases requiring medication were excluded. Women with normal pregnancies were matched for maternal age, BMI, living habits, and medical history. A total of 455 mothers and their neonates were recruited in this study.

### Meconium collection, DNA extraction, and sequencing

First-pass meconium samples (around 200 mg) were collected on sterilized diapers by well-trained nurses within the first few hours of birth at the labor ward. Meconium samples were stored temporarily in an ice box and transported to the laboratory within 24 h. Thereafter, the samples were stored at −80°C until DNA or metabolite extraction. QIAamp Fast DNA Stool Mini Kit (QIAGEN, Germany) was used to extract the DNA in a decontaminated and sterile environment. Negative controls during sample collection, transportation, and extraction were included and used in the data analysis. The genomic DNA was used as the template to amplify the V3 hypervariable region of the 16S rRNA gene with the forward primer (5′-CCAGACTCCTACGGGAGGCAG-3′) and the reverse primer (5′-CGTATTACCGCGGCTGCTG-3′). The PCR products were checked by agarose gel electrophoresis, and then the PCR product was used as a template, and the index PCR was performed by using index primers for adding the Illumina index to the library. The amplification products were checked using gel electrophoresis and were purified using the Agencourt AMPure XP Kit (Beckman Coulter, CA, USA). The purified products were indexed in the 16S V3 library. The library quality was assessed on the Qubit 2.0 Fluorometer (Thermo Scientific) and Agilent Bioanalyzer 2100 systems. Finally, the pooled library was sequenced on an Illumina MiSeq Sequencer for generating 2×250 bp paired-end reads.

### Sequencing data processing

Raw data were demultiplexed and quality-controlled (*Q* score > 20, read length > 100). A total of 50,908,512 of 16S rRNA clean reads were generated from the 455 samples (mean reads per sample = 111,887; min to max = 20,237–420,428; SD = 60,009). And we obtained 34,269 ± 3430 reads in sequencing negative controls (*n* = 15). After removing singleton, a total of 10422 different OTUs were picked against the RDP database at 97% sequence similarity. Sequencing contaminants (*n* = 33 from 10,422 total OTUs) were identified and removed using the decontam package reads (frequency methods, *P* < 0.5) [18]. The detailed contaminated OTUs are shown in Additional file 1, Table S1. After decontamination, 37 samples were removed due to the low reads number (less than 10000 reads per sample). Finally, 418 mothers (147 women with GDM and 271 normal pregnant women) and their neonates were retained in this study with 36,995,740 (mean reads per sample = 88,507; min to max = 10,007–420,308; SD = 60,644) reads aligned to Ribosomal Database Project (RDP) by mother (Additional file 1, Table S2) [19]. To adjust for differences in reads number, normalization was carried out by rarefying reads to 10,007 per sample. Finally, 7987 OTUs were remained after removing OTUs with an abundance of 0. The detailed rarefied OTU table is shown in Additional file 1, Table S3. PICRUSt2 was used to predict the metagenomes from the OTU data based on 20,000 16S sequences from genomes in the Integrated Microbial Genomes database [20].

### Metabolomic sample preparation of meconium samples

A total of 50 mg of meconium was dissolved in 500 μL of ultrapure water in a 2-mL centrifuge tube at room temperature. Following ultrasonic homogenization for 5 min and centrifugation at 16,000×*g* for 15 min, the supernatant was transferred into a new 2-mL centrifuge tube. Then, methanol (1500μL) was added into the tube. After ultrasonic homogenization for 5 min and centrifugation at 16,000×*g* for 15 min, the supernatant was transferred into a new 2-mL centrifuge tube, vortexed for 30 s, and filtered through a 0.22-μm filter. The target analytes were concentrated under a speed vacuum concentrator and reconstituted for further analysis.

### Metabolomic sample preparation of maternal blood serum samples

During the course of the study, maternal blood samples were collected on the day before delivery. Maternal blood samples were centrifuged immediately to separate serum and then stored in aliquots at −80 °C for further analysis. A total of 40 μL of methanol was added to 10 μL of maternal serum for protein precipitation. After vortexing for 30 s and centrifuging at 16,000×*g* for 20 min, the supernatant was transferred into a 1.5-mL centrifuge tube. The target analytes were concentrated under a speed vacuum concentrator at room temperature and reconstituted for further analysis.

### Metabolomic analysis

Metabolomic analysis was performed on a UPLC Ultimate 3000 system (Dionex, Germering, Germany) coupled to a Q-Exactive mass spectrometer (QEMS) (Thermo Fisher Scientific, Bremen, Germany) in both positive and negative modes simultaneously. The UPLC analysis was carried out with a Hypersil GOLD C18 column (100 mm × 2.1 mm, 1.9 μm) (Thermo Fisher Scientific) with the column temperature being set at 40 °C. A multistep gradient consisted of mobile phase A (0.1% formic acid in ultrapure water) and mobile phase B (acetonitrile (ACN) acidified with 0.1% formic acid) with a flow rate of 0.4 mL/min and a run time of 15 min. The UPLC autosampler temperature was set at 4°C, and the injection volume for each sample was 10 μL. All samples were analyzed in a randomized fashion to avoid complications related to the injection order. MS data were collected by the QEMS equipped with a heated electrospray ionization (HESI) source. For both positive and negative modes, the operating parameters were as follows: a spray voltage of 3.5 kV for positive mode and 2.5 kV for negative mode, a capillary temperature of 300°C, a sheath gas flow of 50 arbitrary units, an auxiliary gas flow of 13 arbitrary units, a sweep gas of 0 arbitrary units, and an S-lens RF level of 60. In the full-scan analysis (70 to 1050*m*/*z*), the resolution was set at 70,000. The MS system was calibrated according to the manufacturer’s instructions. Chemical identification was based on the retention time and accurate mass of commercial standards.

### Statistical analysis

For comparisons between the GDM and control groups, demographic variables were analyzed by *t*-test or Fisher’s exact test. For microbiota data, the alpha diversity indices were compared by the Wilcoxon rank sum test. The weighted unifrac distance metric was used to determine multivariate sample distances and visualized through principal coordinates analysis (PCoA). Permutation-based analysis of variance (PERMANOVA) was used to compute the difference of β-diversity between GDM and control group using the delivery mode as a covariate. Abundance of genera between the two groups was assessed by Metastats, a common tool to find differentially abundant features among groups [21]. Correlation networks of significantly differentiated genera were generated within the GDM and control groups, using Spearman’s correlation (rho < −0.3 or rho > 0.3; FDR corrected *P* < 0.05). Relative proportions of predicted metagenome functions were compared by statistical hypothesis tests with a corrected *P*-value in STAMP 2.1.3. For metabolomics data, orthogonal partial least squares discrimination analysis (OPLS-DA) was applied to distinguish the differences of metabolomic profile by SIMCA-P 13.0 (Umetrics, Umea, Sweden). Pathway analysis for metabolomics data was conducted by MetaboAnalyst (http://www.metaboanalyst.ca/) to predict enriched pathway of differential metabolites.

## Results

### Characteristics of study participants

Data from 418 mothers (147 women with GDM and 271 normal pregnant women) and their neonates from the GDM Mother and Child Study were finally used in this study. The dataset contained 418 meconium samples from neonates and 315 blood samples from mothers. Of these, 418 meconium samples were all used for 16S sequencing and 619 samples (304 meconium samples and 315 blood samples) were used for metabolome profiling. The characteristics of the participants are presented in Table 1. There were no significant differences in maternal age, pre-pregnancy BMI, smoking, drinking status, and second-hand smoking between the two groups. In addition, the characteristics regarding multiparas, abnormal pregnancy history and family history of diabetes, gestational age, and peripartum antibiotics usage in the GDM group were also not significantly different from those in the control group. However, a higher cesarean section (C-section) rate and increased birth weight were observed in the GDM group than in the control group (*P* < 0.001).

**Table 1:** Characteristics of study participants

Variables	GDM group (***n***=147)	Control group (***n***=271)	***P***
***Maternal***			
Age, years (mean ± SD)	29.51±3.79	29.04±4.00	0.244
Pre-pregnancy BMI, kg/m^2^ (mean ± SD)	21.55±2.99	21.20±3.75	0.327
Smoking status [*n* (%)]			
Ever	0 (0.00)	2 (0.74)	0.763
Never	147 (100.00)	269 (99.26)	
Second-hand smoking [*n* (%)]			
Yes	31 (21.09)	75 (27.68)	0.174
No	116 (78.91)	196 (72.32)	
Drinking status [*n* (%)]			
Ever	1 (0.68)	6 (2.21)	0.443
Never	146 (99.32)	265 (97.79)	
Parity [*n* (%)]			
Nulliparae	127 (86.39)	219 (80.81)	0.191
Multiparae	20 (13.61)	52 (19.19)	
Family history of diabetes [*n* (%)]			
Yes	24 (16.33)	35 (12.92)	0.418
No	123 (83.67)	236 (87.08)	
Gestational age, days (mean ± SD)	277.75±7.47	276.37±7.96	0.085
Peripartum antibiotics [*n* (%)]			
Yes	24 (16.33)	27 (9.96)	0.082
No	123 (3.67)	244 (90.04)	
***Neonatal***			
Sex [*n* (%)]			
Boy	74 (50.34)	140 (51.66)	0.876
Girl	73 (49.66)	131 (48.34)	
Delivery status [*n* (%)]			
Vaginal	87 (59.18)	201 (74.17)	0.002
Cesarean	60 (40.82)	70 (25.83)	
BW, g (mean ± SD)	3511.63±425.40	3329.22±347.42	< 0.001

*Abbreviations*: *GDM* gestational diabetes mellitus, *SD* standard deviation, *BMI* body mass index, *BW* birth weight

### Meconium microbiota was different in neonates born to mothers with GDM

**Fig. 1:**
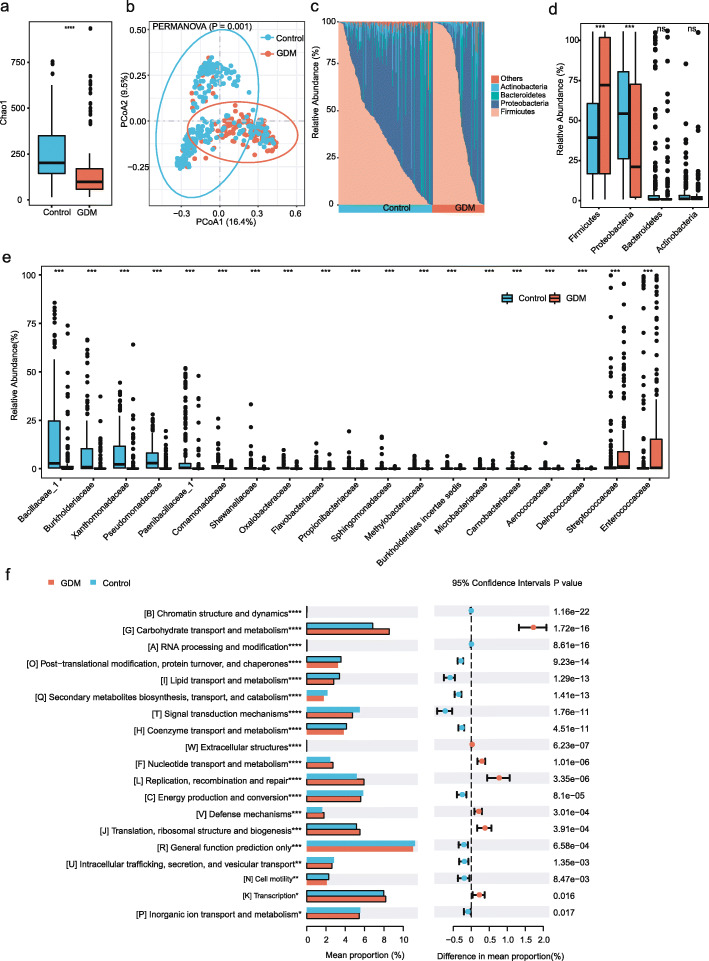
**a** Alpha diversity of the gut microbiome between the two groups. **b** PCoA (based on weighted unifrac distances) of the gut microbiome between the two groups. **c**, **d** Bacterial composition of the meconium at the phylum levels. **e** Relative abundance of differential bacteria (FDR < 0.01) between the two groups. **f** The abundant pathways of microbial genes between the two groups. ***P < 0.001

Our study exhibited a significant reduction in α-diversity (Chao1 index: *P* < 0.001) in neonates born to mothers with GDM when compared with those of mothers without GDM (Fig. 1a). GDM was significantly associated with the shift of β-diversity (*P* = 0.001) (Fig. 1b). At the same time, the β-diversity was significantly different between delivery modes (*P* = 0.011) (Additional file 2, Figure S1). The dominant phyla across all samples were *Proteobacteria*, *Firmicutes*, *Bacteroidetes*, and *Actinobacteria* (Fig. 1c). Compared to the control group, the GDM group showed significant increases in relative abundance of *Firmicutes* and significant decreases in relative abundance of *Proteobacteria* at the phylum level (Fig. 1d). At the family level, the GDM group exhibited more abundances of *Streptococcaceae* (*P* < 0.001), while other families were less abundant in the GDM group than in the control group (Fig. 1e).

Previous studies showed that meconium microbiota was greatly affected by delivery mode [11, 12]. In order to understand whether delivery mode had impacts on meconium microbiota in our study, we further compared the abundance at the family level stratified by delivery modes (vaginal delivery and C-section) (Additional file 2, Figure S2 a,b). Interestingly, we observed bacterial family changing by the similar trend between the different delivery modes, revealing the concordance of microbial variation associated with GDM.

Co-occurrence networks of significantly differentiated genera showed more balanced microbial correlations in the control group than the GDM group (Additional file 2, Figure S3). In the network of the control group, GDM-associated genera such as *Clostridium sensustricto*, *Rothia*, and *Lactobacillus* were negatively correlated with control-associated genera. This was not observed in the network of the GDM group. The difference in co-occurrence networks between the control and GDM groups reflected maternal GDM might disrupt the ecology of meconium microbiota.

In order to understand the potential function, we used PICRUSt2 to predict the metagenome functions [20]. Among the predicted metagenome functions, pathways related to carbohydrate transport and metabolism, nucleotide transport and metabolism, translation, ribosomal structure and biogenesis, and transcription were enriched in the GDM group (Fig. 1f). Pathways related to post-translational modification, protein turnover, and chaperones, lipid transport and metabolism, and signal transduction mechanisms were enriched in the control group (Fig. 1f).

### Meconium metabolome was different in neonates born to mothers with GDM

**Fig. 2:**
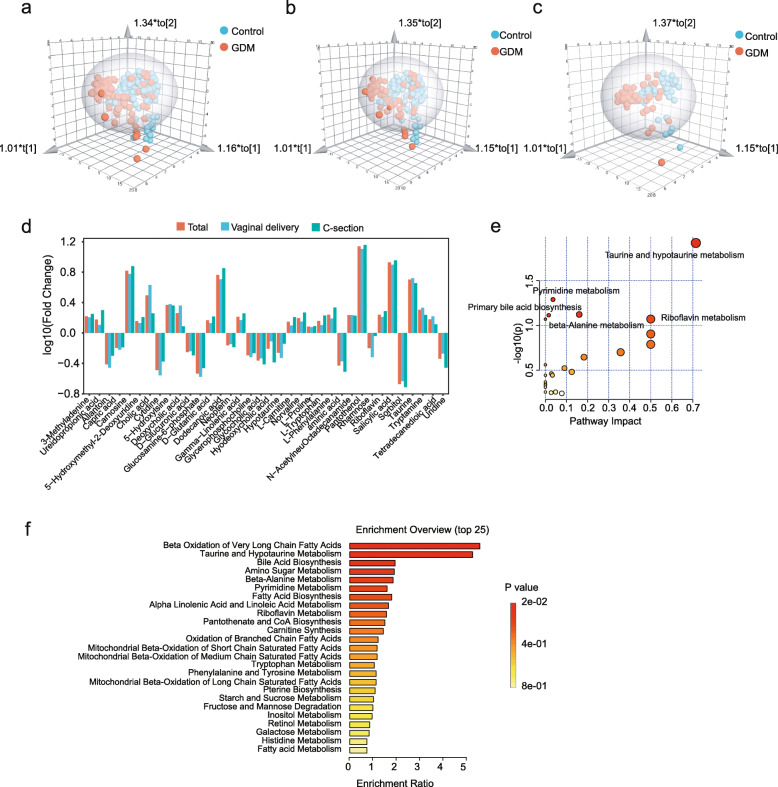
**a**–**c** OPLS-DA models of the meconium metabolomic profiles between the two groups in the whole research population (**a**), vaginal delivery population (**b**), and cesarean section population (**c**). **d** Thirty-six metabolites showed consistent significant changes between the two groups. **e**, **f** Meconium metabolic pathway analysis of differentially abundant metabolites between the two groups

A total of 118 metabolites were detected from 304 meconium metabolomic samples (105 GDM cases and 199 controls). OPLS-DA analyses of meconium metabolites between the two groups were performed based on the whole research population (R^2^X = 0.566, R^2^Y = 0.572, *Q*^2^ = 0.465) (Fig. 2a), vaginal delivery population (R^2^X = 0.569, R^2^Y = 0.554, *Q*^2^ = 0.408), and cesarean section population (R^2^X = 0.536, R^2^Y = 0.511, *Q*^2^ = 0.428) (Fig. 2b, c), indicating that the metabolites in meconium were significantly different between two groups.

Among the identified metabolites, 36 showed consistently significant differences between the two groups in the total population, vaginal delivery population, and cesarean section population (Fig. 2d), indicating that these changes were GDM-related metabolic changes. The abundances of a total of 22 metabolites were significantly increased in the GDM group, including riboflavin and taurine, while those of 14 metabolites were significantly decreased, e.g., glycerophosphocholine (GPC), glycocholic acid, and rhamnose.

Through pathway analysis based on the Kyoto Encyclopedia of Genes and Genomes (KEGG) pathways, we found that these metabolites were enriched in 7 pathways, namely, taurine and hypotaurine metabolism; pyrimidine metabolism; beta-alanine metabolism; bile acid biosynthesis; phenylalanine, tyrosine, and tryptophan biosynthesis; riboflavin metabolism; and aminoacyl-tRNA biosynthesis (Fig. 2e). Through enrichment analysis based on the SMPDB database, we found that taurine and hypotaurine metabolism, pyrimidine metabolism, beta-alanine metabolism, and bile acid biosynthesis were also included in the top 6 pathways (Fig. 2f, Additional file 1, Table S4). These results showed the enrichment significances of metabolic pathways in offspring’s meconium of GDM mothers.

### Serum metabolome was different in mothers with GDM

**Fig. 3:**
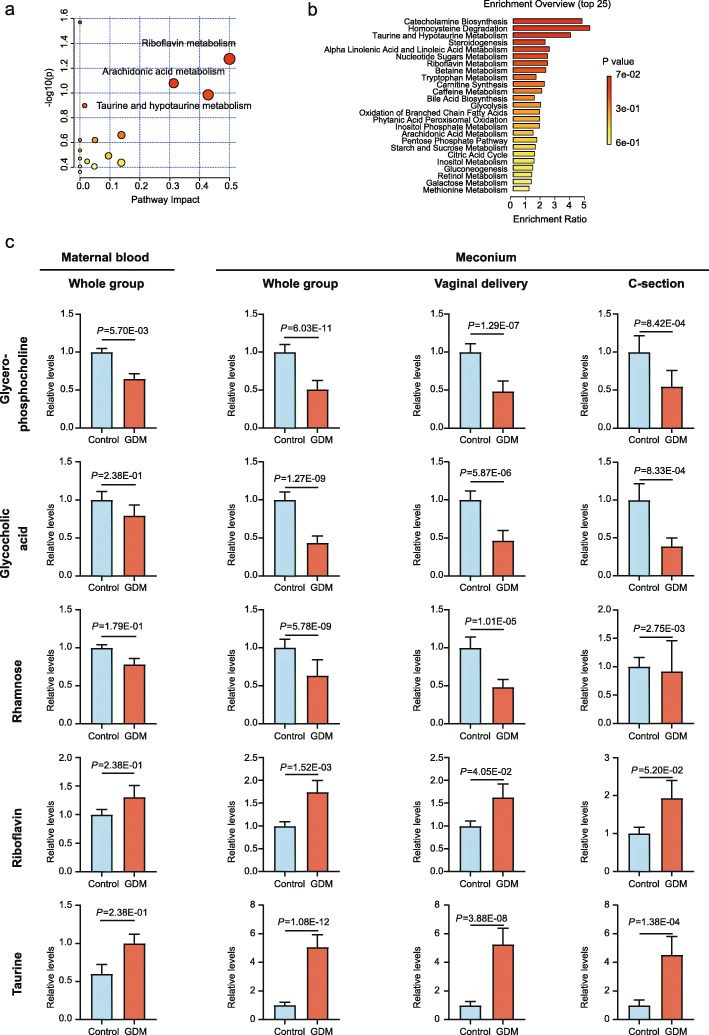
**a** Metabolic pathway analysis by KEGG of maternal blood. **b** Metabolic pathway analysis by enrichment analysis of maternal blood. **c** Key changed metabolite abundances in meconium and maternal blood in relation to GDM. *P < 0.05; **P < 0.01

A total of 113 metabolites were detected in 315 maternal blood samples (59 GDM cases and 256 controls). Twenty-two of them showed significant differences between the two groups. These metabolites were enriched in pathways including riboflavin metabolism, arachidonic acid metabolism, and taurine and hypotaurine metabolism, based on a KEGG pathway analysis (Fig. 3a). In addition, taurine and hypotaurine metabolism was included in the top three pathways in an enrichment analysis based on the SMPDB database (Fig. 3b, Additional file 1, Table S5). These results showed the enrichment significances of metabolic pathways in GDM mothers’ blood samples.

To verify whether the metabolomic profile of meconium was related to the maternal metabolomic profile in relation to GDM, we divided the meconium into the vaginal delivery and C-section groups. Interestingly, we found that five metabolites changed in their abundances in the similar trend in both meconium and maternal blood in relation to GDM. They were GPC, glycocholic acid, rhamnose, riboflavin, and taurine (Fig. 3c). GPC, glycocholic acid, and rhamnose abundances were decreased in the neonatal meconium and maternal blood of the GDM group, while those of riboflavin and taurine were increased.

## Discussion

In this study, we identified the relationship between the meconium microbiota, metabolome in neonates born to mothers with GDM. In addition, we also found five changed metabolites in the similar trend across the meconium and maternal blood. Taken together, our findings illustrated a connection between the bacterial composition and metabolism of neonates, which might be affected by the metabolic state of mothers.

Previous studies reported low richness, low diversity, and a predominance of the phylum *Proteobacteria* in meconium samples [12]. In this study, we also identified a limited number of taxa and *Proteobacteria* as the dominant phylum in the meconium samples. In addition, significantly lower alpha diversity was observed in the GDM group than in the control group. These results were in accordance with decreased microbial richness and diversity of the enteric microbiota in GDM mothers reported previously [22, 23]. Moreover, decreased richness of the enteric microbiota has been associated with elevated insulin resistance and proinflammatory markers [24].

In this study, the abundances of the families *Rothia* and *Clostridium sensustricto*, which may contain opportunistic pathogens that might cause enteric infections and childhood metabolic disorders [25], were significantly increased in the GDM neonates revealed by network analysis. In addition, bacterial family changing was in the similar trend when stratified by delivery modes, which indicated the variation of GDM-related bacteria was consistent. However, the consistency of changed bacteria in neonates might dramatically be altered within days based on feeding (breast/formula) etc., and further studies on gut microbiota in infancy and childhood should be performed to validate the persistence of our findings.

Using metagenome function prediction, we demonstrated that pathways related to carbohydrate and nucleotide metabolism were enriched, which are associated with metabolic diseases, including macrosomia and hypoglycemia of neonates born to GDM mothers. This indicates that maternal GDM may facilitate the succession of high-energy-providing microbiota with altered metabolism in their children; consequently, this could mediate the development of childhood obesity in later life.

For the five consistently changed metabolites in meconium and maternal blood, the declining trends of GPC, glycocholic acid, and rhamnose in mothers with GDM and their neonates were consistent with previous studies [26–29]. GPC is a subclass of the glycerophospholipids and is associated with lipid metabolism, whereas glycocholic acid is related to the digestion and absorption of lipids. These results indicate that GDM may be associated with altered lipid and carbohydrate metabolism in neonates, possibly mediated by maternal blood metabolites. Collectively, the five consistently changed metabolites indicated the possible hazardous effect of GDM on children via the disruption of metabolism, which may be through GDM mothers. In addition, in the pathway analysis of the metabolomes, similar to the findings in the microbiota meconium analysis, nucleotide metabolism pathways (pyrimidine metabolism) were also enriched in the metabolomic profile of meconium of the GDM group, indicating the importance of this nucleotide change in neonates in relation to GDM.

One strength of our study is that it contains both mothers and their neonates, allowing us to investigate potential trans-generation effects of GDM. Another strength is that we performed sensitivity analysis by the mode of delivery in the analysis of the meconium microbiota and meconium metabolome. This implies their associations with GDM without being confounded by birth modes. Despite having these strengths, a causal relationship between the metabolites and microbiota in meconium cannot be confirmed by the cross-sectional study design. As meconium is the sample with low biomass, the method we used to remove OTUs may fail to detect contaminants that are uniformly present in the samples. More effective negative control samples are needed to detect the potential technical contaminants in future studies. In addition, all participants in the current study were Han Chinese. Given that the enteric microbiota varies among different races, further studies should be performed in other populations to validate and extend our findings.

## Conclusions

Our study provided information about the relationships of maternal metabolome, meconium microbiota, and metabolome. We observed that certain meconium metabolites varied in a similar trend with the maternal serum metabolites associated with GDM. These data highlight the importance of understanding the effects of pregnancy complications on the formation of early-life microbiota and metabolome. Further studies are warranted to explore their implications for infant health later in life.

## Supplementary Information

**Additional file 1: Table S1.** Relative abundance of OTUs with contamination. **Table S2.** Bacteria composition in meconium samples (non-rarefied data). **Table S3.** Bacteria composition in meconium samples (rarefied data). **Table S4.** Metabolic pathway of differentially abundant metabolites related to GDM in meconium. **Table S5.** Metabolic pathway of differentially abundant metabolites related to GDM in maternal blood.

**Additional file 2: Figure S1.** PCoA (based on weighted unifrac distances) of the gut microbiome by delivery mode. **Figure S2.** The abundances of the dominant families between two groups when stratified by delivery mode (a: vaginal delivery, b: C-section). **Figure S3.** Correlation networks of significantly differentiated genera in neonates born to control mothers (A) and GDM mothers (B). Red and green edges represented positive and negative correlations, respectively. Yellow and blue nodes indicate genera enriched in the control and the GDM group, respectively. In addition, node size denotes the mean relative abundance of the genus within each group.

## Data Availability

The datasets used and/or analysed during the current study are available from the corresponding author on reasonable request.

## Abbreviations

16S rRNA: 16S ribosomal RNA
ACN: Acetonitrile
BMI: Body mass index
CPR: Computer-based patient record
C-section: Cesarean section
DNA: Deoxyribonucleic acid
FDR: False discovery rate
GDM: Gestational diabetes mellitus
GMCS: GDM Mother and Child Study
GPC: Glycerophosphocholine
HESI: Heated electrospray ionization
IRB: Institutional Review Board
KEGG: Kyoto Encyclopedia of Genes and Genomes
OGTT: Oral glucose tolerance test
OPLS-DA: Orthogonal partial least squares discrimination analysis
OTUs: Operational taxonomic units
PCoA: Principal coordinates analysis
PCR: Polymerase chain reaction
PICRUSt: Phylogenetic Investigation of Communities by Reconstruction of Unobserved States
QEMS: Q-Exactive mass spectrometer
SD: Standard deviation
SMPDB: The Small Molecule Pathway Database
UPLC-QE: Ultra performance liquid chromatography-Q exactive
