# Supplementary figures and images for: Gestational diabetes mellitus is associated with the neonatal gut microbiota and metabolome
Source: BMC Med. 2021 May 27;19:120. doi: 10.1186/s12916-021-01991-w (PMC8157751; doi:10.1186/s12916-021-01991-w)

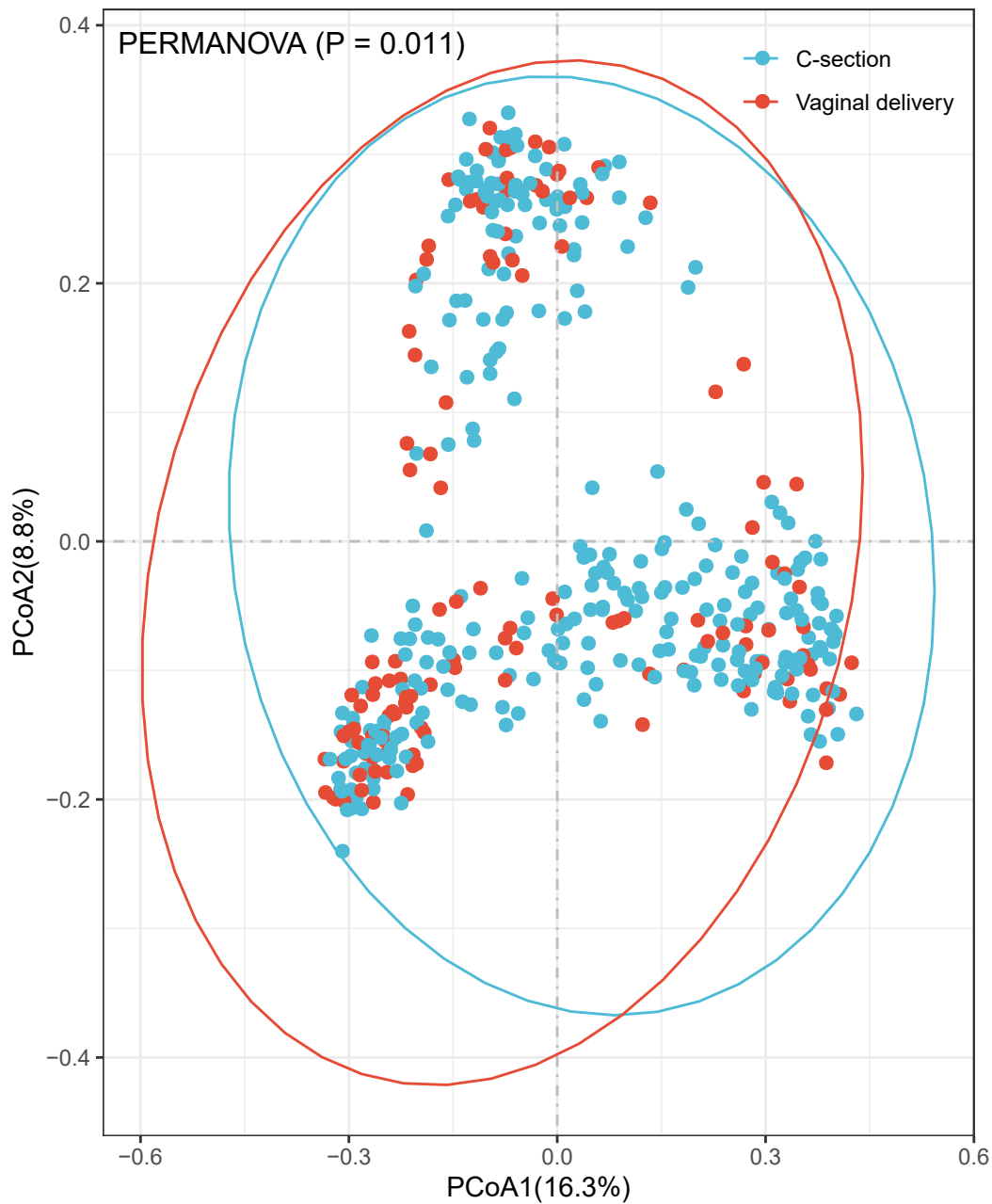

a

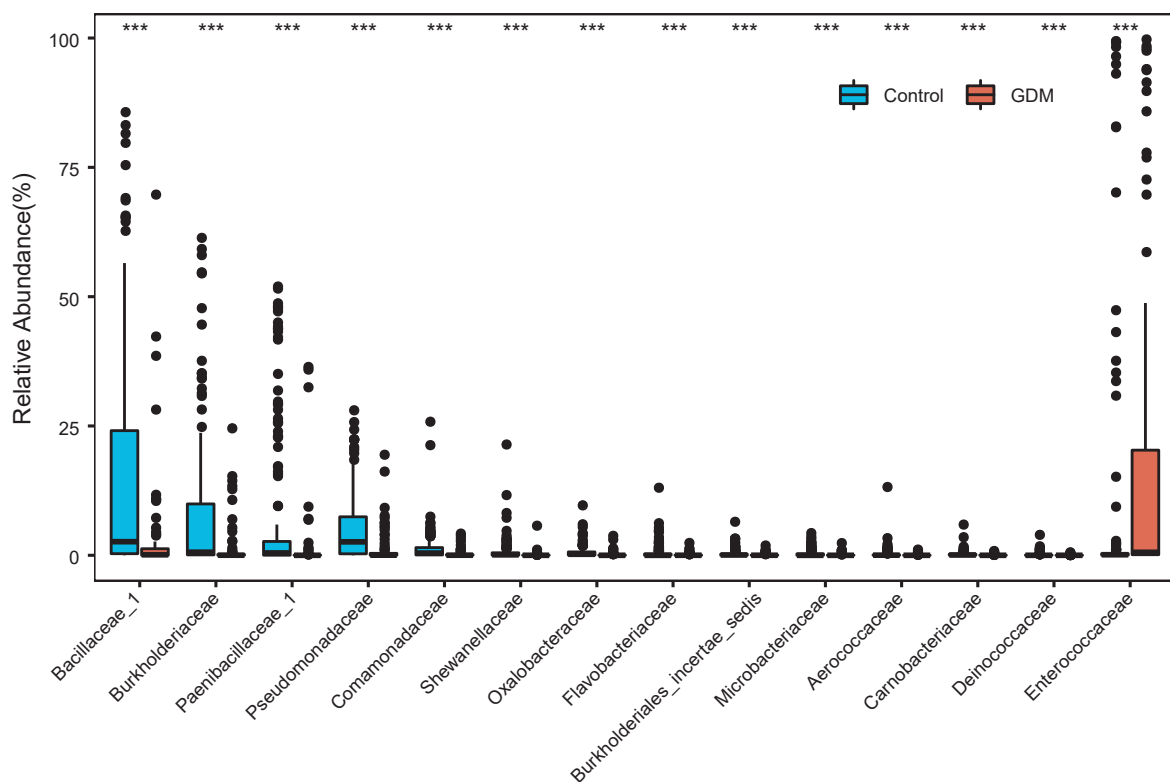

b

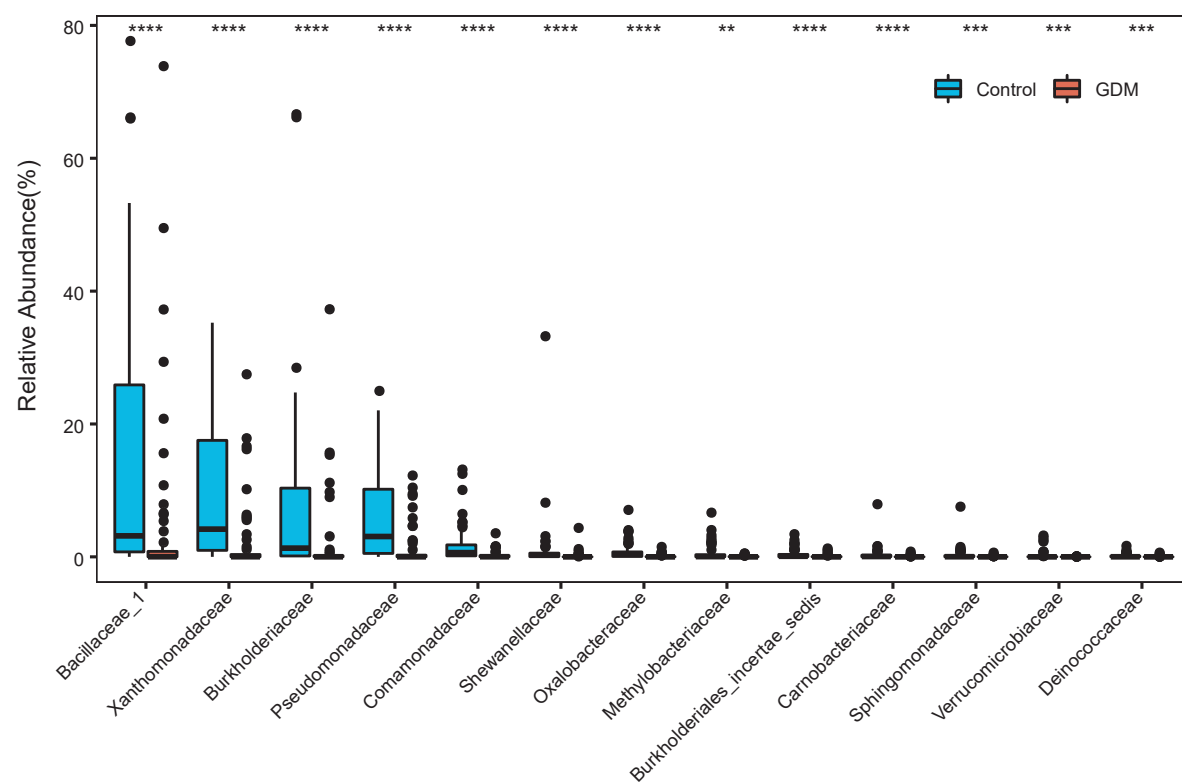

a

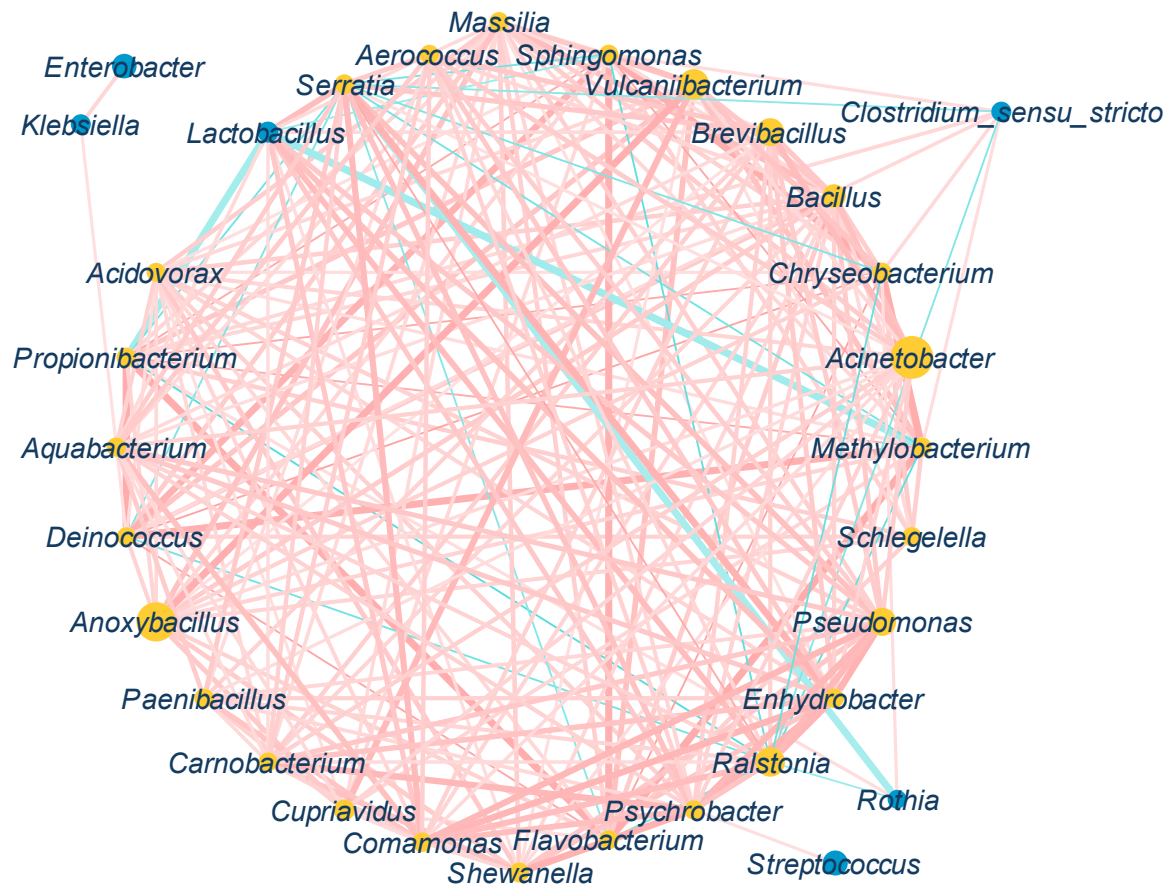

b

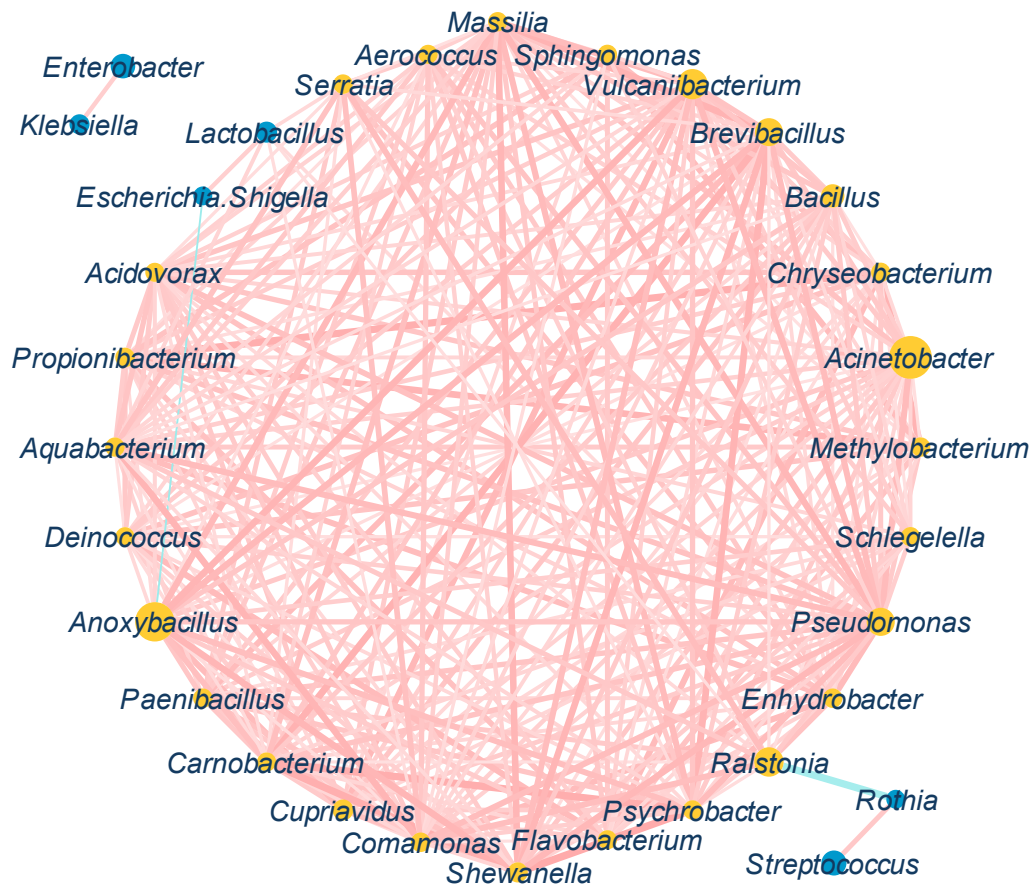

Supplement: Supplementary file 2 — Additional file 2: Figure S1. PCoA (based on weighted unifrac distances) of the gut microbiome by delivery mode. Figure S2. The abundances of the dominant families between two groups when stratified by delivery mode (a: vaginal delivery, b: C-section). Figure S3. Correlation networks of significantly differentiated genera in neonates born to control mothers (A) and GDM mothers (B). Red and green edges represented positive and negative correlations, respectively. Yellow and blue nodes indicate genera enriched in the control and the GDM group, respectively. In addition, node size denotes the mean relative abundance of the genus within each group. [file 12916_2021_1991_MOESM2_ESM.pdf]
